# Bioproduction of raspberry ketone using *Phyllostachys* bamboo cells expressing raspberry ketone biosynthetic genes

**DOI:** 10.5511/plantbiotechnology.25.0627a

**Published:** 2025-12-25

**Authors:** Takao Koeduka, Keisuke Yoshida, Taiji Nomura

**Affiliations:** 1 Graduate School of Sciences and Technology for Innovation, Yamaguchi University, 1677-1 Yoshida, Yamaguchi, Yamaguchi 753-8515, Japan; 2 Biotechnology Research Center and Department of Biotechnology, Toyama Prefectural University, 5180 Kurokawa, Imizu, Toyama 939-0398, Japan

**Keywords:** bamboo cell culture, bioconversion, 4-hydroxybenzalacetone, raspberry ketone

## Abstract

Bioproduction of high-value natural compounds in cultured plant cells represents a favorable strategy compatible with the framework of green sustainable chemistry. Raspberry ketone, a phenylpropanoid-derived compound utilized as a food additive and cosmetics constituent, is a distinctive aromatic component that is accumulated in the ripe fruit of raspberry (*Rubus idaeus*). Its natural abundance is extremely limited. Consequently, it is imperative to develop methodologies for efficient bioproduction of raspberry ketone. In the present study, we examined the effect of expressing the raspberry ketone biosynthetic genes *RpBAS* and *RiRZS1* in bamboo (*Phyllostachys nigra*) cells, which had been demonstrated previously to be an appropriate host for production of phenylpropanoid-derived compounds, on the bioconversion of precursor compounds into raspberry ketone. The maximal production yield of raspberry ketone, primarily accumulated in the glycosylated form, reached 293 µg g^−1^ fresh weight when the *RpBAS*-*RiRZS1*-transgenic bamboo cells were cultured in medium supplemented with 4-hydroxybenzalacetone, the immediate precursor of raspberry ketone. These findings underscore the potential utility of bamboo cells for the bioproduction of raspberry ketone from accessible precursors.

Raspberry ketone [4-(4-hydroxyphenyl)butan-2-one] is a key volatile compound contributing to the characteristic aroma of the ripe fruit of raspberry (*Rubus idaeus*). The compound has been extensively utilized as a fragrance component in perfumes, cosmetics, and air fresheners; however, its natural abundance is extremely low, including in raspberry fruit (1–4 mg kg^−1^ fruit) (Lee 2016). The chemical synthesis of raspberry ketone via the aldol condensation of *p*-hydroxybenzaldehyde with acetone has been reported (Malkar and Yadav 2019). Nevertheless, owing to regulatory constraints, such synthetic products cannot be marketed as “natural” flavoring agents under the prevailing food legislation. Natural flavors include compounds produced through microbial or enzymatic processes using precursors that occur naturally or are obtained via biotechnological methods. Consequently, the biosynthetic pathway of raspberry ketone and its associated enzymes have been elucidated, prompting numerous reports of metabolic engineering of microbial and plant systems aimed at enhancing its biosynthesis (Häkkinen et al. 2015; Koeduka et al. 2021; Masuo et al. 2022). The development of efficient and scalable production strategies remains a critical objective.

Production of a target natural compound, which is originally present in the mother plant, using cultured cells is one viable alternative to extraction from plant resources and chemical synthesis. However, no practical strategy for raspberry ketone production using cultured cells of raspberry has yet been established (Häkkinen et al. 2015). In addition, cultured plant cells may be used to produce a target compound that is not originally present in the mother plant, which requires the creation of transgenic plant cells. Although such synthetic biology-oriented heterologous production of a target compound in plant cell hosts has not been pursued extensively, a “rational metabolic-flow switching” strategy has been demonstrated to be an effective approach for this purpose (Nomura et al. 2018).

Through a series of proof-of-concept studies, it has been demonstrated that cultured cells of a bamboo species (*Phyllostachys nigra*; Pn) are a suitable host for the bioproduction of phenylpropanoid-derived compounds, i.e., hydroxycinnamoylagmatines, 4-hydroxybenzoic acid derivatives, and 4-vinylphenol derivatives, via rational metabolic-flow switching (Kitaoka et al. 2020, 2021; Nomura et al. 2018; Ube et al. 2024). These successful examples were achieved by redirecting the highly active inherent metabolic pathways of Pn cells, i.e., hydroxycinnamoylputrescines and lignin (Nomura et al. 2013; Ogita et al. 2012) (Figure 1), to the related pathways that originate from the key intermediate compounds hydroxycinnamoyl-CoAs, such as *p*-coumaroyl-CoA and feruloyl-CoA. Given that raspberry ketone is biosynthesized from *p*-coumaroyl-CoA and malonyl-CoA (Koeduka et al. 2021), Pn cells are considered a suitable host for heterologous production of raspberry ketone. In the present study, we generated transgenic Pn cells that express two crucial enzymes for raspberry ketone biosynthesis, namely, *Rheum palmatum* benzalacetone synthase (RpBAS) and *Rubus idaeus* raspberry ketone/zingerone synthase 1 (RiRZS1), to enable raspberry ketone production. As a strategy to achieve elevated synthesis of raspberry ketone, we demonstrate the effectiveness of supplementation of the culture medium with 4-hydroxybenzalacetone, the immediate precursor of raspberry ketone. The study results contribute to development of a practical bioproduction system for raspberry ketone in cultured plant cells.

**Figure 1:**
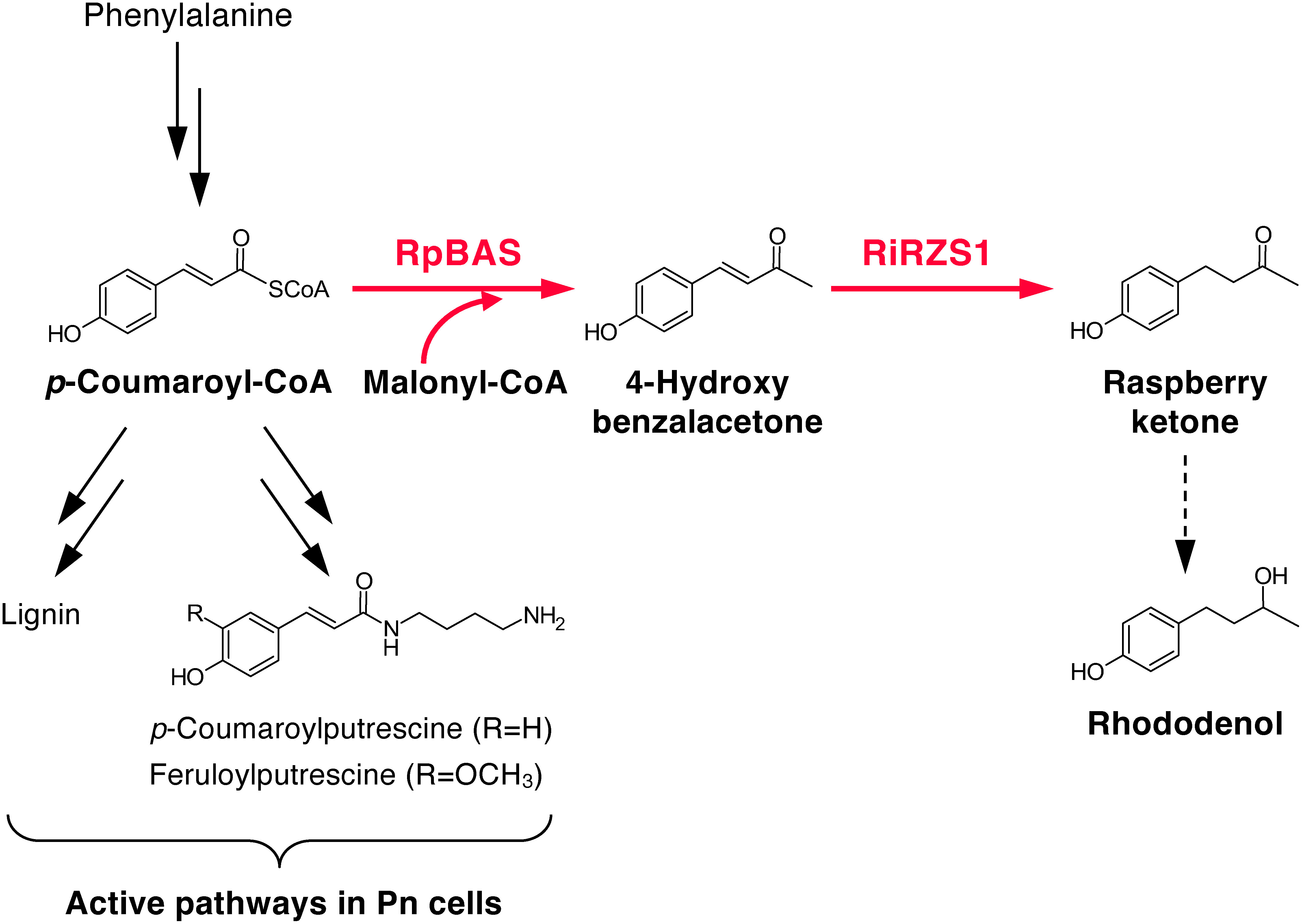
Figure 1. Schematic diagram of metabolic engineering of the endogenous phenylpropanoid pathway in Pn cells for raspberry ketone biosynthesis by introducing benzalacetone synthase (RpBAS) and raspberry ketone/zingerone synthase 1 (RiRZS1).

To generate stable transgenic Pn cell lines expressing the *RpBAS* and *RiRZS1* genes (GenBank accession nos. AF326911 and JN166691, respectively), the binary vector pRI201-ON-Hm (Kitaoka et al. 2020), harboring *HPT* (hygromycin phosphotransferase) and *NPTII* (neomycin phosphotransferase II) as selectable markers, was employed as the expression vector backbone. The coding sequences of *RpBAS* and *RiRZS1*, flanked by the *Nde*I and *Sal*I or *Xho*I restriction sites (Koeduka et al. 2021), were independently ligated into the *Nde*I/*Sal*I-digested pRI201-ON-Hm vector. The *RiRZS1* expression cassette, comprising the Cauliflower mosaic virus 35S promoter, *Oryza sativa* alcohol dehydrogenase 5′-untranslated region, and *Arabidopsis thaliana* heat shock protein terminator, was amplified by PCR and subsequently inserted downstream of the *RpBAS* expression cassette into a *Kpn*I-linearized *pro35S:RpBAS* construct (Supplementary Figure S1) using the In-Fusion HD Cloning Kit (Takara Bio, Shiga, Japan) following the manufacturer’s protocol. The resulting construct, pRI201-ON-Hm/RpBAS-RiRZS1, was introduced into Pn suspension cells via particle bombardment using the Biolistic Particle Delivery System (Bio-Rad, Hercules, CA, USA) as previously described (Ogita et al. 2011).

Transformed Pn cells were grown on proliferation (PR) medium solidified with 0.3% (w/v) gellan gum–modified Murashige and Skoog (MS) medium (Murashige and Skoog 1962) supplemented with 680 mg l^−1^ KH_2_PO_4_, 10 µM picloram (4-amino-3,5,6-trichloropyridine-2-carboxylic acid), and 3% (w/v) sucrose in the dark at 25°C, and subsequently, the cells were transferred to selective solid PR medium supplemented with 100 mg l^−1^ hygromycin B, following the protocol of Nomura et al. (2018). Ten hygromycin B-resistant calli were selected as candidate transformants. Total RNA was extracted from the calli using previously established methods (Koeduka et al. 2021). The introduction and expression of the *RpBAS*, *RiRZS1*, and *HPT* transgenes were confirmed by reverse transcription PCR (RT-PCR) using gene-specific primers (Supplementary Table S1), using the housekeeping gene *actin* as an internal reference. PCR amplification was conducted in a 10 µl reaction mixture comprising first-strand cDNA, 0.1 µM each primer, and 2× EmeraldAmp MAX PCR Master Mix (Takara Bio). The thermal-cycling protocol comprised initial denaturation at 98°C for 10 s, followed by 35 cycles of denaturation at 98°C for 10 s, primer-specific annealing at 56°C for 30 s, and extension at 72°C for 1 min, respectively. Amplification products for *RpBAS*, *RiRZS1*, *HPT*, and *actin* were detected by agarose gel electrophoresis (Supplementary Figure S2). Two independent transgenic lines (#4 and #5) exhibiting active proliferation, hereafter designated TL1 and TL2, were selected for subsequent experiments and cultured in liquid PR medium as suspension cells. The TL1 and TL2 cells were subcultured at 4-week intervals until stable proliferation was achieved.

For the bioproduction of raspberry ketone in TL1 and TL2 cells, 2-week-old subcultured suspension cells grown in PR medium were transferred to 30 ml fresh half-strength MS medium containing 3% (w/v) sucrose in a 100 ml Erlenmeyer flask with an initial sedimented cell volume adjusted to 10%. This medium, designated LG1 (Nomura et al. 2013), has been demonstrated to activate phenylpropanoid metabolism, including hydroxycinnamoylputrescines and lignin (Figure 1). As previously reported (Koeduka et al. 2021), raspberry ketone biosynthesis is catalyzed by RpBAS and RiRZS1 from *p*-coumaroyl-CoA and malonyl-CoA, when these precursors are sufficiently available in the transformants. However, raspberry ketone and its glycosylated derivatives were not detected in the transgenic Pn cells over the 14-day culture period (data not shown), although both transgenes were moderately expressed (Supplementary Figure S2). Considering that Pn cells cultured in LG1 medium actively biosynthesize hydroxycinnamoylputrescines, which are formed by enzymatic condensation of hydroxycinnamoyl-CoA and putrescine, and lignin, which is a polymer of three monolignols that are biosynthesized from *p*-coumaroyl-CoA (Nomura et al. 2013, 2018; Ogita et al. 2012), the supply of *p*-coumaroyl-CoA to RpBAS, the first-committed enzyme for raspberry ketone biosynthesis, is likely to be sufficient. However, no secondary metabolites that are derivatives of malonyl-CoA, such as anthocyanins, have been detected in Pn cells. We thus predicted that the absence of raspberry ketone and its derivatives in the transgenic Pn cells is due to insufficient quantities of endogenous malonyl-CoA for raspberry ketone production. However, supplementation with dimethylmalonate and sodium malonate to the culture medium did not lead to the detection of raspberry ketone or its glycosylated derivatives. Moreover, we could not detect the enzyme activity of RpBAS, forming 4-hydroxybenzalacetone from *p*-coumaroyl-CoA and malonyl-CoA, in the crude extract of TL1 or TL2. These facts indicate that the rate of translation of *RpBAS* transcripts is limited in Pn cells. To elevate the translation efficiency, introduction of modified *RpBAS* sequence whose codon is optimized to the monocotyledonous type may be required.

Accordingly, a bioconversion assay was conducted employing the immediate precursor, 4-hydroxybenzalacetone, to facilitate raspberry ketone biosynthesis in the transgenic Pn cells expressing *RpBAS* and *RiRZS1*. Pn cells transformed with the empty vector pRI201-ON-Hm were used as the negative control. To evaluate the impact of varying the precursor concentration, the transgenic Pn cells were cultured in LG1 medium for 24 h, as described above. Subsequently, 4-hydroxybenzalacetone was added to the culture medium at a final concentration of 0.05, 0.10, or 0.15 mg ml^−1^. After culture for 24 h, the cells were harvested by vacuum filtration and subsequently subjected to metabolite analysis as described below.

Volatile aglycons were extracted from approximately 0.5 g fresh weight (FW) of the cells using 4 ml methyl *tert*-butyl ether as the solvent. For analysis of glycosylated volatiles, approximately 0.5 g FW of the cells were frozen in liquid nitrogen then ground with a mortar and pestle. The resulting fine powder was suspended in 3 ml of 80% (v/v) methanol and sonicated for 20 min. After centrifugation at 500×g for 15 min, the supernatant containing glycosylated volatiles was transferred to a clean glass tube and subjected to enzymatic hydrolysis, following the protocol described by Koeduka et al. (2021). The resulting extract was analyzed using a GC-2010 Plus gas chromatography-mass spectrometry (GC-MS) system (Shimadzu Corporation, Kyoto, Japan) equipped with a DB-5 ms capillary column (30 m length, 0.25 mm inner diameter, 0.25 µm film thickness; Agilent Technologies, Santa Clara, CA, USA). The GC temperature program was set as follows: initial temperature of 80°C held for 1 min, increased at 2°C min^−1^ to 160°C, then at 20°C min^−1^ to 240°C, and held at 240°C for 2 min. Compound identification, including 4-hydroxybenzalacetone, raspberry ketone, and rhododenol, was conducted by comparing retention times and mass spectra with those of authentic standards.

Raspberry ketone (18–53 µg g^−1^ FW) and its glycosides (140–183 µg g^−1^ FW) were detected in the transgenic lines TL1 and TL2 cultured in the presence of 0.05 mg ml^−1^ 4-hydroxybenzalacetone, whereas substantially higher contents of glycosides (212–238 µg g^−1^ FW) under supplementation with 0.10 or 0.15 mg ml^−1^ 4-hydroxybenzalacetone were detected (Figure 2). In addition, trace amounts of glycosylated rhododenol, a downstream metabolite derived from raspberry ketone (Figure 1), were detected in the transgenic lines cultured in the presence of 0.10 mg ml^−1^ 4-hydroxybenzalacetone (Supplementary Figures S3, S4). In the control Pn cells transformed with the empty vector, 44–58 µg g^−1^ FW raspberry ketone glycosides were detected under supplementation with 0.05 or 0.10 mg ml^−1^ 4-hydroxybenzalacetone (Figure 2). These results suggest that, although weak olefin reductase activity toward 4-hydroxybenzalacetone is endogenously present in Pn cells, the conversion of 4-hydroxybenzalacetone to raspberry ketone was significantly enhanced in the transgenic Pn cells by the activity of RiRZS1. Meanwhile, supplementation with a high concentration of 4-hydroxybenzalacetone (e.g., 0.15 mg ml^−1^) resulted in lower levels of production of raspberry ketone and its glycosides compared to supplementation at 0.05 or 0.10 mg ml^−1^ (Figure 2B). This reduction may be due to cytotoxic effect of 4-hydroxybenzalacetone at a high concentration, which could vary depending on cell line-specific characteristics.

**Figure 2:**
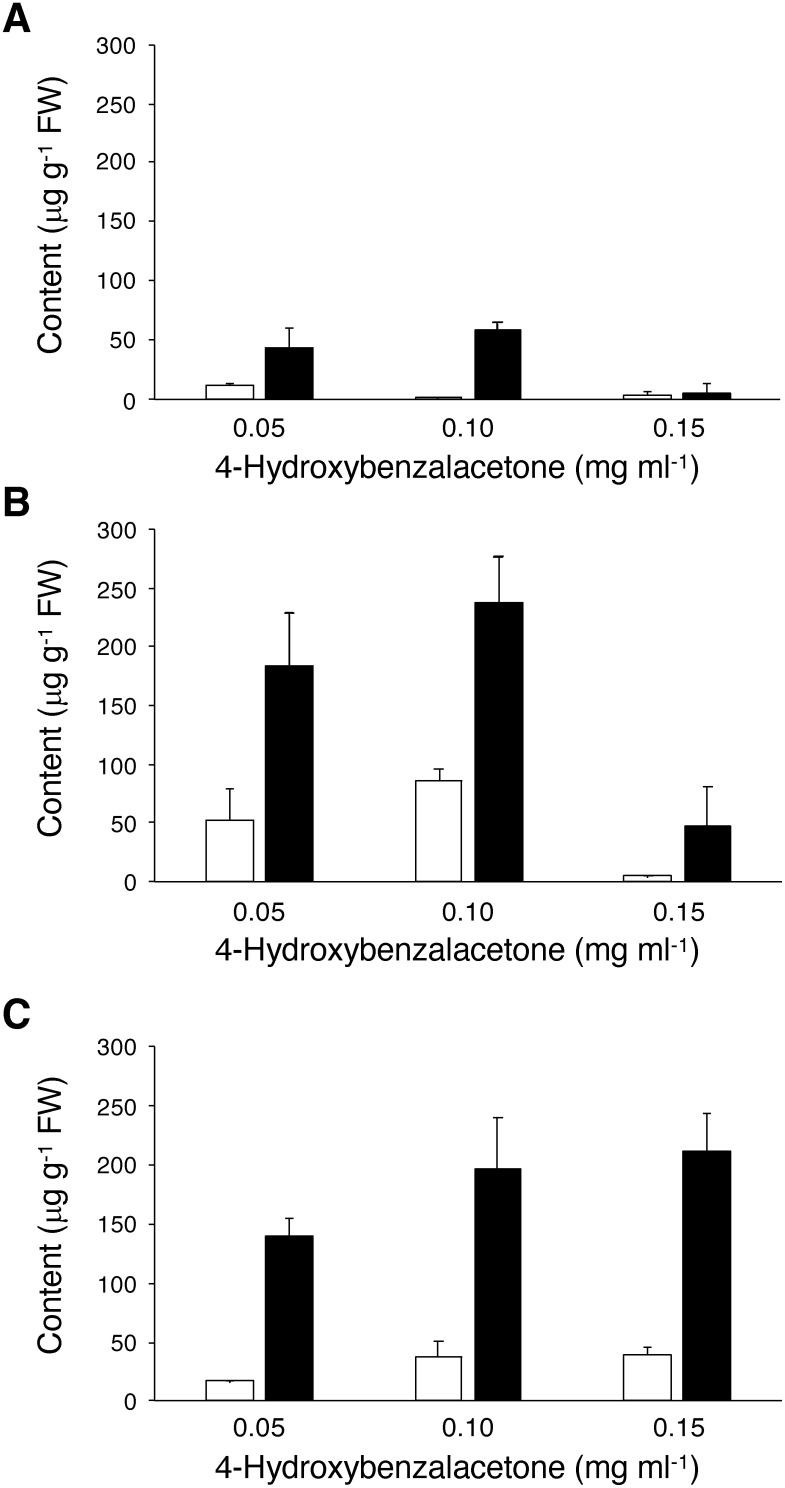
Figure 2. Raspberry ketone production in transgenic Pn cells cultured for 24 h in the presence of different concentrations of 4-hydroxybenzalacetone. (A) Vector control, (B) *RpBAS*-*RiRZS1* transgenic line 1 (TL1), (C) *RpBAS*-*RiRZS1* transgenic line 2 (TL2). Empty bars, raspberry ketone aglycon; filled bars, raspberry ketone glycoside. Data are means±SD (*n*=3).

Next, we examined the temporal changes (at 3, 12, 24, and 48 h) in raspberry ketone production in the transgenic Pn cells cultured in the presence of 0.10 mg ml^−1^ 4-hydroxybenzalacetone. Low contents of raspberry ketone (5–14 µg g^−1^ FW) were detected after culture for 12 h, which increased 3- to 10-fold during culture for 24–48 h (Figure 3). The content of raspberry ketone glycosides peaked at 282–293 µg g^−1^ FW during culture for 12–24 h and gradually declined up to 48 h. In contrast, only trace amounts of raspberry ketone and modest contents of its glycosides (32–76 µg g^−1^ FW) were detected in the control Pn cells transformed with the empty vector.

**Figure 3:**
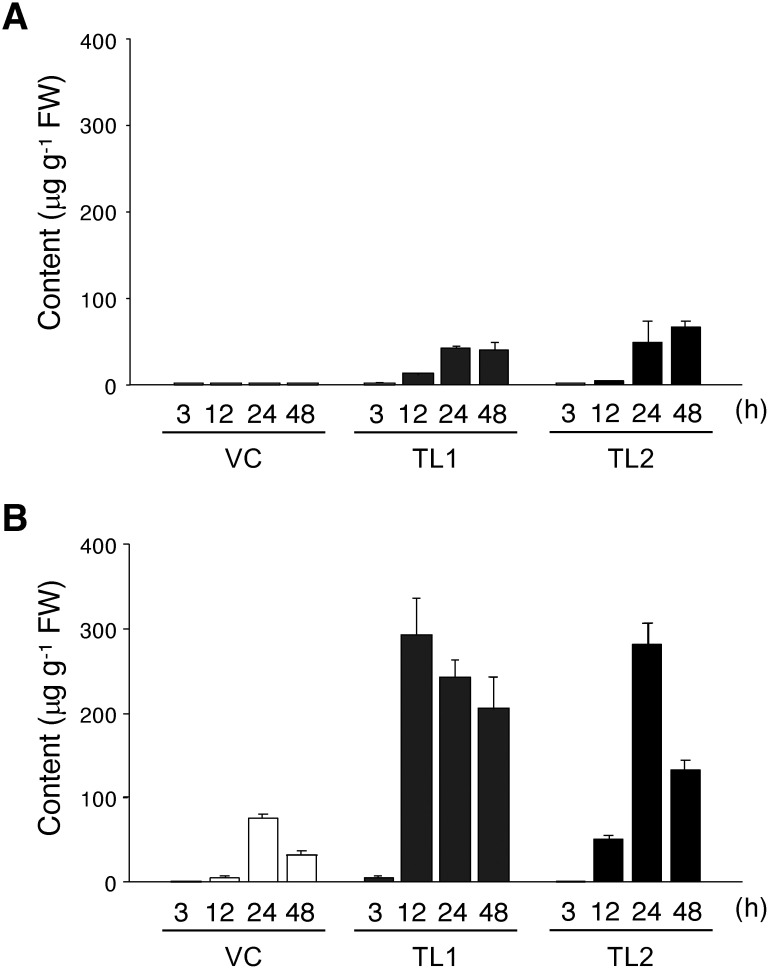
Figure 3. Effect of culture period on raspberry ketone production in transgenic Pn cells cultured for 3, 12, 24, and 48 h in the presence of 0.10 mg ml^−1^ 4-hydroxybenzalacetone. (A) Raspberry ketone aglycon, (B) raspberry ketone glycoside. Empty bars, vector control (VC); gray bars, *RpBAS*-*RiRZS1* transgenic line 1 (TL1); filled bars, *RpBAS*-*RiRZS1* transgenic line 2 (TL2). Data are means±SD (*n*=3).

In the present study, the transgenic Pn cells that expressed the raspberry ketone biosynthetic genes, *RpBAS* and *RiRZS1*, unexpectedly failed to efficiently produce raspberry ketone and its glycosides in the absence of supplementation with the exogenous precursor. However, upon supplementation with 0.10 mg ml^−1^ 4-hydroxybenzalacetone, the transgenic Pn cells accumulated substantial quantities of raspberry ketone and its glycosides, with maximum titers of 67 µg g^−1^ FW for aglycon (TL2, 48 h) and 293 µg g^−1^ FW for glycosides (TL1, 12 h). The aglycon and glycoside yields correspond to approximately 742 and 3250 µg g^−1^ dry weight, respectively. Although the substrate-to-product conversion efficiencies (1.1% for aglycone and 5.8% for glycoside) were not so high, the maximum titers are more than five-times higher than the previously reported titers that were achieved by bioconversion of exogenously supplied 4-hydroxybenzalacetone in cultured cells of various plant species (Häkkinen et al. 2015).

In light of the increasing demand for natural flavor compounds such as raspberry ketone, bioconversion by cultured plant cells represents a promising and efficient approach for the transformation of precursor substrates into high-value metabolites. Consequently, the bioconversion of 4-hydroxybenzalacetone utilizing transgenic Pn cells is a valuable contribution to the biotechnological production of natural raspberry ketone. This system is viable because commercial 4-hydroxybenzalacetone is reasonably available. Nevertheless, bioproduction of raspberry ketone without precursor supply is preferable. This may be achieved by introduction of the gene for acetyl-CoA carboxylase, which catalyzes the formation of malonyl-CoA from acetyl-CoA, to the transgenic Pn cells expressing *RpBAS* and *RiRZS1*, which can be predicted to overcome the shortage of malonyl-CoA supply to RpBAS. Furthermore, utilizing cultured plant cells that possess a highly active anthocyanin biosynthetic property as a host for heterologous expression of *RpBAS* and *RiRZS1* may be an effective alternative, because such cells can actively produce *p*-coumaroyl-CoA and malonyl-CoA, the starting substrates for anthocyanin biosynthesis. Redirection of these two compounds to raspberry ketone biosynthesis is considered a rational approach. Verification of these alternative strategies by our research group is currently in progress.

## Abbreviations

Pn: *Phyllostachys nigra*
RiRZS1: *Rubus idaeus* raspberry ketone/zingerone synthase 1
RpBAS: *Rheum palmatum* benzalacetone synthase

## Description of supplementary files

Supplementary Table S1. Primer sequences used in this study.

Supplementary Figure S1. Schematic diagram of the expression cassette for raspberry ketone biosynthetic genes.

Supplementary Figure S2. RT-PCR analysis for detection of transcripts of raspberry ketone biosynthetic genes in transformed Pn calli.

Supplementary Figure S3. GC-MS analysis of volatile metabolites extracted from transgenic Pn cells.

Supplementary Figure S4. Mass spectra of raspberry ketone and rhododenol detected by GC-MS analysis.
