# Supplementary material for: Bioproduction of raspberry ketone using Phyllostachys bamboo cells expressing raspberry ketone biosynthetic genes
Source: Plant Biotechnol (Tokyo). 2025 Dec 25;42(4):497–501. doi: 10.5511/plantbiotechnology.25.0627a (PMC12781914; doi:10.5511/plantbiotechnology.25.0627a)
Supplement: Supplementary Data [file plantbiotechnology-42-4-25.0627a-s001.pdf]

Supplementary Information

for:

Bioproduction of raspberry ketone using *Phyllostachys* bamboo cells expressing raspberry ketone biosynthetic genes

Takao Koeduka[1], Keisuke Yoshida[1], Taiji Nomura[2,*]

[1]*Graduate School of Sciences and Technology for Innovation, Yamaguchi University, 1677-1 Yoshida, Yamaguchi, Yamaguchi 753-8515, Japan*

[2]*Biotechnology Research Center and Department of Biotechnology, Toyama Prefectural University, 5180 Kurokawa, Imizu, Toyama 939-0398, Japan*

[*]Corresponding author e-mail: tnomura@pu-toyama.ac.jp

Supplementary Table S1. Primer sequences used in this study.

Direction	Sequence (5′ to 3′)
For RT-PCR analysis	
For *RpBAS*	
Forward	CATATGGCTACTGAAGAGATGAAG
Reverse	TCAAGAGATCACTGGCACAG
For *RiRZS1*	
Forward	GCGCGGCAGCCATATGGCGAGTGGTGGAGAAATG
Reverse	ACGGAGCTCGAATTCCTCGAGTCACTCTCTGGAAACAACCAC
For *HPT*	
Forward	ATGAAAAAGCCTGAACTCACC
Reverse	CTATTCCTTTGCCCTCGGAC
For *actin*	
Forward	GGTATACGCTTCCTCACGCT
Reverse	CTCGCAGTCTCAAGCTCCT
For vector construction using In-Fusion cloning	
Forward	AAAGATAAGGAATTGGTCCCCAGATTAGCCTTTT
Reverse	ATCCAGCGGCCGCAGAATTCCTTATCTTTAATCATATTC

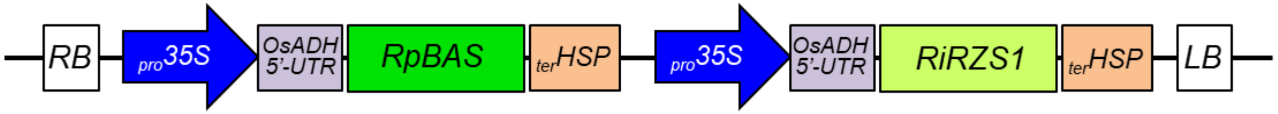

Supplementary Figure S1. Schematic diagram of the expression cassette for raspberry ketone biosynthetic genes. *pro35S*, Cauliflower mosaic virus 35S promoter; *OsADH 5′-UTR*, 5′-untranslated region of *Oryza sativa alcohol dehydrogenase*; *RpBAS*, *Rheum palmatum benzalacetone synthase*; *RiRZS1*, *Rubus idaeus raspberry ketone/zingerone synthase 1*; *terHSP*, terminator of *Arabidopsis thaliana* heat shock protein.

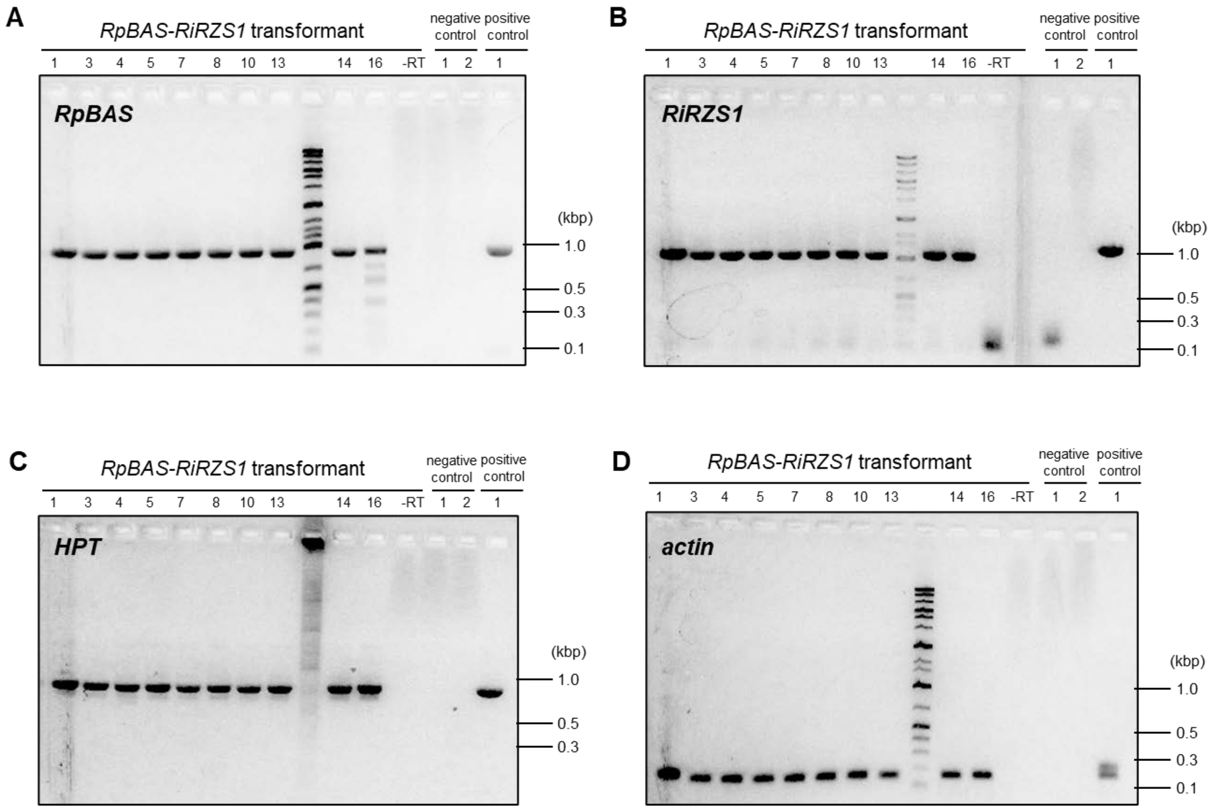

Supplementary Figure S2. RT-PCR analysis for detection of transcripts of raspberry ketone biosynthetic genes in transformed Pn calli. Total RNA was extracted from the vector control and *RpBAS–RiRZS1*-transformed Pn calli. PCR amplification of *RpBAS* (A), *RiRZS1* (B), *HPT* (C), and *actin* (D, internal reference gene) was performed using first-strand cDNA as the template. For the positive control, plasmid DNA (for *RpBAS*, *RiRZS1*, and *HPT*) or genomic DNA (for *actin*) was used as the template. The PCR mixture without template/primer was used as the negative control.

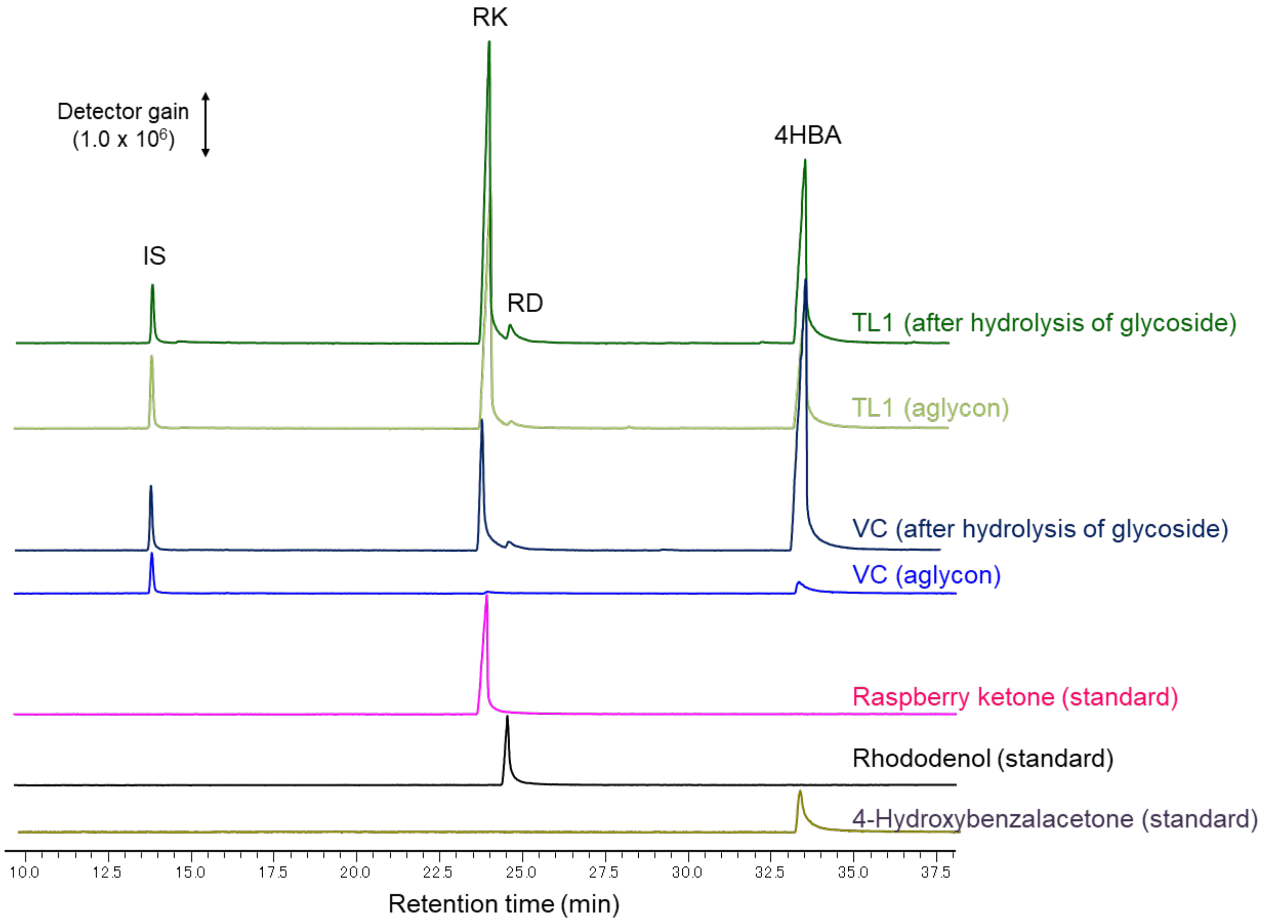

Supplementary Figure S3. GC-MS analysis of volatile metabolites extracted from transgenic Pn cells.
TL1, transgenic line 1; VC, vector control; IS, internal standard (dihydroeugenol); RK, raspberry ketone; RD, rhododenol; 4HBA, 4-hydroxybenzalacetone.

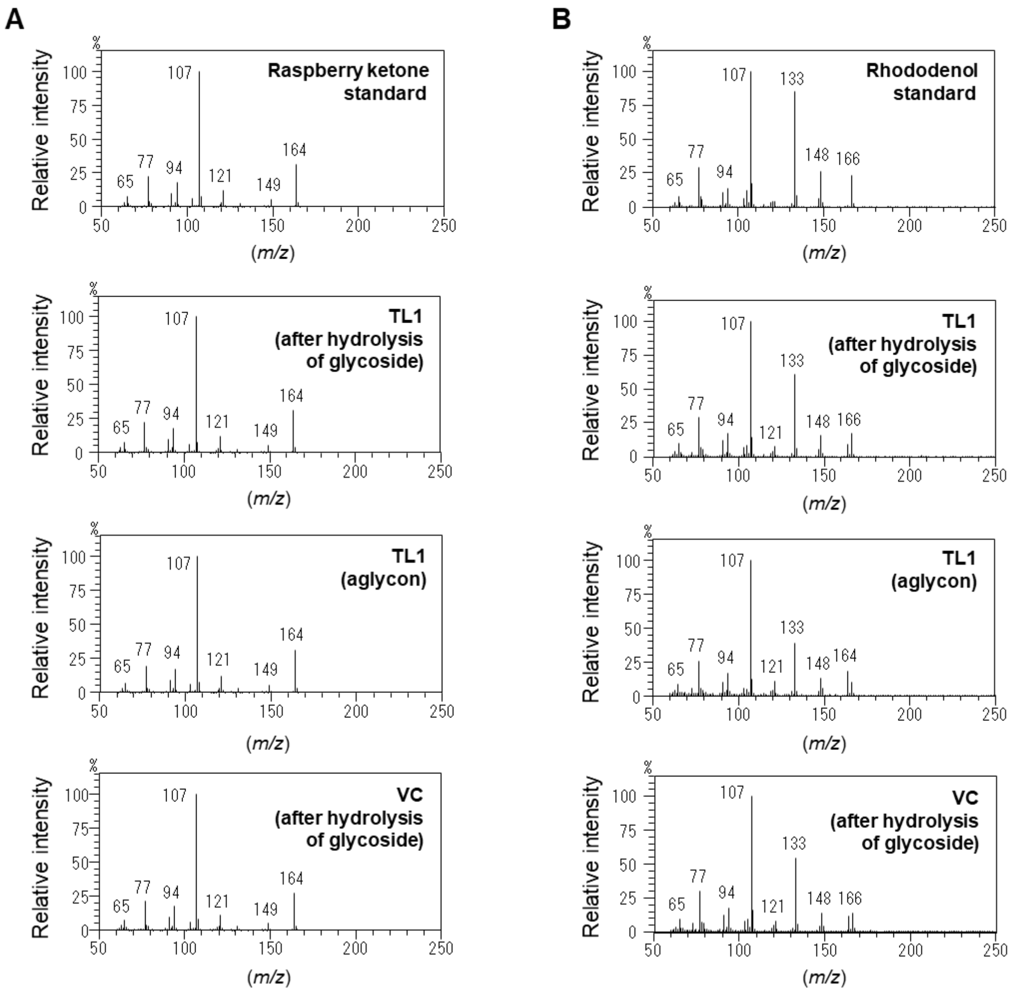

Supplementary Figure S4. Mass spectra of raspberry ketone (A) and rhododenol (B) detected by GC-MS analysis. TL1, transgenic line 1; VC, vector control.
